# *In Vitro* Evaluation of Wound Healing, Stemness Potentiation, Antioxidant Activity, and Phytochemical Profile of *Cucurbita moschata* Duchesne Fruit Pulp Ethanolic Extract

**DOI:** 10.1155/2024/9288481

**Published:** 2024-10-28

**Authors:** Preeyaporn Plaimee Phiboonchaiyanan, Saraporn Harikarnpakdee, Thanapat Songsak, Verisa Chowjarean

**Affiliations:** ^1^Department of Pharmacology, College of Pharmacy, Rangsit University, Pathum Thani 12000, Thailand; ^2^Department of Industrial Pharmacy, College of Pharmacy, Rangsit University, Pathum Thani 12000, Thailand; ^3^Department of Pharmacognosy, College of Pharmacy, Rangsit University, Pathum Thani 12000, Thailand; ^4^Department of Pharmaceutical Technology, College of Pharmacy, Rangsit University, Pathum Thani 12000, Thailand

**Keywords:** antioxidant, *Cucurbita moschata*, migration, pumpkin, stem cell, wound healing

## Abstract

Wound healing comprises an intricate process to repair damaged tissue. Research on plant extracts with properties to expedite wound healing has been of interest, particularly their ability to enhance the stemness of keratinocyte stem cells. Hence, the present study aims to determine the wound healing and stemness potentiation properties of an ethanolic extract derived from *Cucurbita moschata* fruit pulp (PKE). Human keratinocytes (HaCaT) and primary skin fibroblast cells were used in this study. The migration of the cells was examined by using a scratch wound healing assay, and spheroid behavior was determined by using a spheroid formation assay. The proteins related to migration and stemness were further measured by using Western blotting to explore the mechanism of action of PKE. The methods used to evaluate PKE's antioxidant properties were 2,2-diphenyl-2-picrylhydrazyl (DPPH) scavenging, ABTS radical scavenging activity, and superoxide anion radical scavenging (SOSA) assays. The phytochemistry of the PKE was investigated using phytochemical screening and high-performance liquid chromatography (HPLC) analysis. The results of this study indicate that nontoxic concentrations of PKE increase the rate of migration and spheroid formation. Mechanistically, PKE increased the expression of the migratory-related protein active FAK (phosphorylated FAK), and the subsequence increased the level of p-AKT. The expression of stem cell marker CD133, upstream protein signaling *β*-catenin, and self-renewal transcription factor Nanog was increased. The PKE also possessed scavenging properties against DPPH, ABTS, and SOSA. The phytochemistry analyses exhibited the presence of alkaloids, glycosides, xanthones, triterpenes, and steroids. Additionally, bioactive compounds such as ɑ-tocopherol, riboflavin, protocatechuic acid, *β*-carotene, and luteolin were detected. The presence of these chemicals in PKE may contribute to its antioxidant, stem cell potentiation, and wound-healing effects. The findings could be beneficial in the identification of valuable natural resources that possess the capacity to be used in the process of wound healing through the potentiation of stemness via a readily detectable molecular mechanism.

## 1. Introduction

The healing process of a wound consists of four well-defined stages: hypercoagulation and vasoconstriction, acute inflammation, cellular proliferation, and final wound remodeling [1]. Platelet thrombosis and fibrin clots are consequences of the coagulation process, which then trigger an inflammatory response by attracting neutrophils and macrophages. The production of growth factors and proinflammatory cytokines stimulates cells involved in the antimicrobial response. The cells included in this group are keratinocytes, endothelial cells, and fibroblasts. Cells release reactive oxygen species (ROS) during inflammatory responses as a protective physiological response to infections. Fibroblasts commence the cellular growth phase by producing extracellular matrix (ECM) components, such as collagen and fibronectin. The process of re-epithelialization and tissue remodeling occurs thereafter [1]. In this context, keratinocytes induce fibroblasts to generate growth factors that regulate their proliferation [2]. The impairment of wound healing may be caused by an excessive generation of ROS, infections, or uncontrolled inflammation. Elevated levels of ROS lead to keratinocyte cellular damage and impaired wound healing [1]. An abundance of ROS can degrade the ECM protein and the biological functions of fibroblasts and keratinocytes [3]. A condition that includes antioxidants may accelerate the wound-healing process [4].

Stemness refers to the inherent ability of cells to undergo both self-renewal and differentiation. These cells have the potential to overcome the limits of traditional wound care techniques by promoting faster tissue regeneration during wound healing [5, 6]. Keratinocyte stem cells play a crucial role in maintaining the balance and healing processes of the skin [5]. Accordingly, a possible approach to improving tissue balance during wound healing is to enhance the functionality and number of keratinocyte stem cells [5, 6]. Studies in the area of stem cell biology have shown the essential role of several proteins, including CD133 and *β*-catenin, in maintaining the properties of stem cells [7, 8]. CD133, a cell surface marker, has been shown to regulate cellular growth, development, and activities associated with the maintenance and function of stem cells [7]. *β*-Catenin influences both the stem cell self-renewal and differentiation processes as it is a co-transcription factor of T-cell factor/lymphoid enhancing factor (TCF/LEF) [8]. Nanog, a transcription factor responsible for self-renewal, potentially contributes to the maintenance of stem cells and interacts with other essential proteins that control pluripotency [9]. Keratinocytes show significant potential as primary sources of skin stem cell factor, and HaCaT cells serve as a valuable model for forthcoming investigations on stem cell factors generated from keratinocytes [5]. Accordingly, one potential approach to achieving enhanced tissue homeostasis throughout the wound-healing process could involve augmenting the functionality of keratinocyte stem cells.

Keratinocytes are the first cells to migrate to the wound site, commence re-epithelialization, and enhance cell proliferation, eventually occupying the wound area throughout the healing process [1]. The focal adhesion kinase (FAK) pathway, essential for the structuring and movement of cells, has been shown to have a function in controlling the assembly of filamentous actin (F-actin) and focal adhesions. Studies have provided evidence indicating that FAK plays a role in cell migration [10]. Specifically, migratory keratinocytes during epidermal wound healing entering a damaged monolayer in vitro have been seen to display increased FAK expression [11]. Current evidence suggests that FAK controls cellular morphology and movement via ATP‐dependent tyrosine kinase (AKT) and other downstream pathways [11].

Vegetable pumpkins belong to the Cucurbitaceae family. Plants that may be cultivated from this family, comprising approximately 800 species and 130 genera, are distributed worldwide [12]. The presence of bioactive compounds in many parts of pumpkins, including their seeds, fruits, and peel, has attracted significant attention due to their potential benefits for nutrition and health. A diverse array of pharmacological activities have been reported, including antioxidant [13], analgesic and anti-inflammatory [14], antibacterial [15], anticarcinogenic [16], antidiabetic and antihyperlipidemic [17], and immunomodulatory capabilities [18]. A vital component of pumpkin is its fruit as it includes essential nutritional elements and bioactive compounds such as carotenoids, phenolic acids, amino acids, vitamins, and minerals [13, 19, 20]. *Cucurbita moschata* pulp is rich in carotenoids (e.g., *β*-carotene, lutein, and zeaxanthin) and phenolic acids (e.g., gallic acid and protocatechuic acid), indicating the presence of an antioxidant effect [13, 21]. Flavonoids, such as quinic acid, p-coumaric acid, cirsiliol, and luteolin, are also present [22]. The fruit pulp of *C. moschata* contains vitamins and minerals such as *α*-tocopherol, vitamin B1, riboflavin, vitamin C, potassium, calcium, and sodium [13, 19, 23]. *C. moschata* has previously demonstrated wound-healing properties in animal models [24, 25]. Shaygan et al. found that hydroalcoholic extractions of *C. moschata* Duchesne fruit peel improved wound healing in a rat model of excision wound repair. The effects were attributed to a reduction in serum nitrite concentration [24]. The ethanolic extract derived from the fruit peel of *C. moschata* Duchesne has been shown to enhance wound contraction in rat skin burns. The tissue indicators associated with oxidative stress also exhibit a reduction [25]. Despite the plant's medicinal potential and its abundance of phytochemicals, no studies have been undertaken to examine the impact of *C. moschata* Duchesne on migration regulatory protein FAK/AKT expression and the stemness of human keratinocytes.

Hence, the present study aimed to assess the antioxidant potential, chemical composition, and ability to promote keratinocyte stem cell function and wound healing of an ethanolic extract derived from *Cucurbita moschata* fruit pulp (PKE). This assessment was conducted in vitro using human keratinocytes and primary skin fibroblast cells. Additionally, possible signaling pathways that govern the migration and stemness of keratinocytes were discovered. The findings may potentially uncover valuable natural resources that may be used in the therapy of wound healing through an increment of stemness via an obviously identifiable molecular mechanism.

## 2. Material and Methods

### 2.1. Collection of Pumpkins, Extraction Procedure, and Chemical Reagents

**Figure 1:**
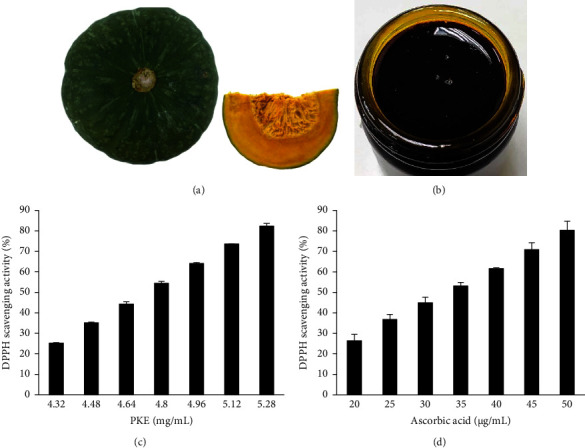
(a) *Cucurbita moschata* Duchesne (Cucurbitaceae). (b) The ethanolic extract from pumpkin meat. Assessment of the DPPH radical scavenging abilities of (c) PKE and (d) ascorbic acid at various concentrations. Data are shown as the mean ± SD (*n* = 3).

Figure 1(a) shows the origin of the pumpkins used in this study as Doi Kham Food Products Co. Ltd., established under the Royal Project initiated by His Majesty, King Bhumibol Adulyadej of Thailand. A botanist from the Department of Pharmacognosy at the College of Pharmacy at Rangsit University, Thailand, confirmed the authenticity of the sample by comparing it to a herbarium specimen labeled with the accession number BKF: 156050. The pumpkins were subjected to an exhaustive procedure of washing, peeling, and slicing before being placed in a preheated oven set at a temperature of 45°C for the purpose of drying. The desiccated fruit of the pumpkins was crushed and underwent three cycles of maceration in ethanol (1:9 w/v) at a temperature of 25°C. Then they were filtered, and the liquid was subjected to evaporation under vacuum pressure at temperatures lower than 40°C. Sigma-Aldrich Company (St. Louis, USA) provided the analytical grade chemicals and solvents.

### 2.2. Determination of Antioxidant Activities

#### 2.2.1. 2,2-Diphenyl-2-picrylhydrazyl (DPPH) Assay

The PKE's ability to scavenge the DPPH radical was assessed using the DPPH assay [26]. The samples were mixed in a 96-well plate with DPPH in ethanol at a concentration of 0.15 mM. The mixture was incubated at room temperature, in the absence of light, for a duration of 30 min. An absorption measurement was taken using a UV spectrophotometer set at 517 nm. The data were represented by calculating the percentage of DPPH inhibition. The vehicle, both with and without DPPH solution, was used as a control for background subtraction. The negative control was the solution without the DPPH solution. The positive control was ascorbic acid (vitamin C).

#### 2.2.2. ABTS Radical Scavenging Activity

180 *μ*L of ABTS•+ solution was mixed with 20 *μ*L of PKE in a 96-well plate [26]. After mixing the solutions using a shaker, they were thereafter allowed to incubate at ambient temperature for a duration of 5 min in the absence of light. The solution was analyzed for absorbance at a wavelength of 750 nm using a UV-spectrophotometer. In comparison to the negative control, which included an ABTS•+ solution mixed with the vehicle, the results were reported as the percentage of ABTS•+ inhibition by PKE. The calculation of the IC_50_ value for a 50% decrease in ABTS+ was performed. Trolox was used as the positive control in this experiment.

#### 2.2.3. Superoxide Anion Radical Scavenging (SOSA) Determination

An equal amount of PKE extract (20 *μ*L) was mixed with phosphate buffer (20 *μ*L), NADH (80 *μ*L), NBT (80 *μ*L), and PMS (20 *μ*L) in a 96-well plate [26]. Then they were incubated at room temperature in darkness for a duration of 15 min. The absorbance of both mixtures was quantified at a wavelength of 560 nm using a UV spectrophotometer. The results were shown as the percentage of SOSA inhibition by PKE in relation to the blank solution as a negative control. The IC_50_ value has been determined for reducing superoxide anion radicals by 50%. This experiment used ascorbic acid as the positive control.

### 2.3. Determination of Total Phenolic Content

The Folin–Ciocalteu test was used to evaluate the total phenolic content of PKE [26]. The samples were mixed with the reagent on a 96-well plate, followed by the addition of Na_2_CO_3_. The absorbance at 765 nm was measured using a UV spectrophotometer after incubating for 1 h. The phenolic content of the PKE was evaluated in gallic acid equivalents (GAEs/g of PKE) using a gallic acid standard curve. The experiment was conducted three times.

### 2.4. Phytochemical Screenings

The presence of secondary metabolites, including steroids, anthraquinones, glycosides, tannins, xanthones, triterpenes, and alkaloids, was examined in PKE [27]. The color scale was used to categorize them as either unidentified (−), low (+), moderate (++), or high (+++) amounts.

#### 2.4.1. Alkaloid Content

0.025 g of PKE was added to a solution of 2N HCl. Centrifugation was used to separate the solutions after the heating of the mixture in a water bath. Subsequently, the solution was incubated with a few drops of Dragendorff's reagent. The occurrence of a reddish-brown precipitate was seen as an indication of the presence of alkaloids.

#### 2.4.2. Anthraquinone Content

0.025 g of PKE was added to a solution of benzene. Following the collection of the benzene layer into the test tube using centrifugation, a solution of NaOH was added. A pink coloration served as an indication of the presence of anthraquinones.

#### 2.4.3. Glycoside Content

A solution containing 0.025 g of PKE in water was incubated in a water bath. The solution was obtained by the process of centrifugation and was applied as a spot on a thin-layer chromatography (TLC) plate. The composition of the mobile phase was butanol/acetic acid/diethyl ether/water (9:6:1:3). The detection process included the use of a solution consisting of 10% sulfuric acid (v/v) and the application of heat. A brown spot at the same level as the reference standard served as an indication of the presence of glycosides.

#### 2.4.4. Tannin Content

A solution was prepared by completely dissolving 0.025 g of PKE in 98% (v/v) ethanol. The solutions were separated using centrifugation after mixing with a vortex mixer. A 10% (v/v) solution of ferric chloride was thereafter added drop by drop. The existence of tannins was verified by the production of a precipitate that varied in color from blue to black.

#### 2.4.5. Xanthone Content

A solution containing 0.025 g of PKE and methanol was incubated in a water bath. The solutions were separated using centrifugation. Subsequently, 5% potassium hydroxide was added. The yellow coloration serves as an indication of the existence of xanthones.

#### 2.4.6. Triterpene Content

0.025 g of PKE was dissolved in dichloromethane. The solutions were separated using the process of centrifugation, and 96% (v/v) sulfuric acid was added. The presence of triterpenes was indicated by a brown ring.

#### 2.4.7. Steroid Content

A solution was prepared by dissolving 0.025 g of PKE in chloroform. The solutions were separated using the process of centrifugation. Subsequently, a solution containing 96% (v/v) sulfuric acid and acetic acid was introduced. A yellow color showed the presence of triterpenes.

### 2.5. Bioactive Compound Determination by High-Performance Liquid Chromatography (HPLC)

#### 2.5.1. Phenolic Compounds and Riboflavin Content

The extract was dissolved in DMSO and then analyzed using HPLC. A diode array detector (DAD) and an Inertsil ODS C18 column (5 *μ*m, 4.6 mm × 150 mm) were used in conjunction with a UV detector on an Agilent 1260 series HPLC system (Santa Clara, CA, USA) to measure the concentrations of luteolin, protocatechuic acid, and riboflavin. The sample was introduced into a 20-*μ*L solution by injection. The gradient elution was carried out at 35°C with acetonitrile and acetic acid as a mobile phase at a flow rate of 1.0 mL/min. For UV detection, the wavelength was optimized for phenolic compounds and riboflavin at their maximum absorbance wavelengths (luteolin at 350 nm, protocatechuic acid at 254 nm, and riboflavin at 270 nm). Each compound was identified based on the retention duration of standard compounds. Their components were measured using calibration curves of standard solutions of riboflavin, protocatechuic acid, and luteolin. The experiment was carried out in triplicate.

#### 2.5.2. *β*-Carotene Content

The extract was dissolved in DMSO and then analyzed using HPLC. A DAD and an Eclipse XDB C18 column (5 *μ*m, 4.6 mm × 150 mm) were used in conjunction with a UV detector (450 nm) on an Agilent 1260 series HPLC system (Santa Clara, CA, USA) to measure the concentrations of *β*-carotene. The sample was introduced into a 20-*μ*L solution by injection. The gradient elution was carried out at 35°C with water and acetone as a mobile phase at a flow rate of 1.0 mL/min. *β*-Carotene was identified by comparing its retention time with that of reference compounds. The *β*-carotene concentration was measured using calibration curves of standard solutions. The experiment was carried out in triplicate.

#### 2.5.3. *α*-Tocopherol Content

The extract was dissolved in DMSO and then analyzed using HPLC. A DAD and an ACE Generix 5 C18 column (5 *μ*m, 4.6 mm × 250 mm) were used in conjunction with a UV detector (292 nm) on an Agilent 1260 series HPLC system (Santa Clara, CA, USA) to measure the concentrations of ɑ-tocopherol. The sample was introduced into a 20-*μ*L solution by injection. The isocratic elution was carried out at 25°C with methanol and acetonitrile as a mobile phase at a flow rate of 1.2 mL/min. *α*-Tocopherol was identified by comparing its retention time with that of reference compounds. The *α*-tocopherol concentration was measured using calibration curves of standard solutions. The experiment was carried out in triplicate.

### 2.6. Cell Culture

The human keratinocyte HaCaT cells were provided by the Cell Lines Service (CLS) (CLS, Heidelberg, Germany). The American Type Culture Collection (ATCC) in Manassas, Virginia, USA, supplied the human primary skin fibroblast cells. The cells were grown using Dulbecco's modified Eagle's medium (DMEM). The medium was enriched with 10% fetal bovine serum (FBS), 100 U/mL penicillin, 100 *μ*g/mL streptomycin, and 2 mM L-glutamine. The cells were subjected to controlled conditions, including a high humidity level, a temperature of 37°C, and a carbon dioxide concentration of 5%. A solution containing 0.25% trypsin and 0.53 mM EDTA was used for subculturing cells. Gibco (Grand Island, NY, USA) served as the provider of several substances including media, trypsin, penicillin/streptomycin, L-glutamine, FBS, and phosphate-buffered saline (PBS). The bovine serum albumin (BSA) and propidium iodide (PI) used for this experiment were provided by Sigma-Aldrich (St. Louis, MO, USA). Hoechst 33342 was bought from Life Technologies Corporation (Eugene, OR, USA). The primary antibodies (FAK, phosphorylated FAK, AKT, phosphorylated AKT, CD133, *β*-catenin, Nanog, and GADPH) and secondary antibodies used in this study were provided by Cell Signaling (Danvers, MA, USA).

#### 2.6.1. Cell Viability Assay

The MTT technique was used to perform a cell viability experiment [28]. A cell density of 1 × 10^4^ cells was used to cultivate the cells overnight in 96-well plates. Cells were then treated with PKE doses ranging from 0 to 1000 *μ*g/mL for 24 h. Subsequently, the PKE solutions were eliminated, and the cells were subjected to incubation with MTT at a concentration of 400 *μ*g/mL at a temperature of 37°C for a duration of 4 h in the presence of 5% CO_2_. DMSO was used to dissolve the formazan crystal. The intensity of the purple solution was measured using spectrophotometry at a wavelength of 570 nm. A comparison between the viability (expressed as a percentage) of the cells that were treated with PKE and the viability of the control group was presented.

#### 2.6.2. Cell Death Detection Assay

Cells were treated with PKE as described in the Cell Viability Assay section. The cells were exposed to 10 *μ*g/mL of Hoechst 33342 for 30 min and then subjected to 5 *μ*g/mL of PI for 5 min. Fluorescent microscopy revealed the presence of cells exhibiting DNA breakage and nuclear condensation, as well as necrotic cells that were positive for PI staining. The experiment used a fluorescence microscope (Olympus IX81) in equipment with an Olympus DP72 camera (Olympus, Tokyo, Japan).

#### 2.6.3. Scratch Wound Healing Assay

We used the scratch wound healing test to ascertain the enhancing impact of PKE on the migration of human keratinocyte and primary skin fibroblast cells [29]. Cells (4 × 10^4^ cells) were plated in a 96-well culture plate overnight. A pipette tip (20–200 *μ*L) was used to eliminate the cells that had adhered to the middle of the well. Cells were then treated with PKE (0–100 *μ*g/mL) for 24 h. Bright-field microscopy was used to capture images of the wound regions at 0, 9, 12, and 24 h. The size of the wound regions was quantified using ImageJ software. The migration of cells was measured at certain time intervals relative to the starting time.

#### 2.6.4. Three-Dimensional Spheroid Formation Assay

The effect of PKE on stem cell behavior was investigated using a spheroid formation assay [30]. The cells were incubated with different concentrations of PKE, ranging from 0 to 100 *μ*g/mL, for a duration of 72 h. Subsequently, detachment cells (2.5 × 10^3^ cells) were transferred into an ultra-low attachment plate (24-well plate). Completed DMEM supplemented with fibroblast growth factor (20 ng/mL), epidermal growth factor (20 ng/mL), and insulin (4 mg/mL) was used for spheroid culture. The cells underwent treatment for a duration of 14 days for every 2 days. Olympus IX51 phase-contrast microscopy with an Olympus DP70 camera, Olympus, was visualized on day 14 to observe spheroids.

#### 2.6.5. Western Blot Analysis

The 1 × 10^5^ cells of HaCaT were treated with varying concentrations of PKE (0–100 *μ*g/mL) for 24 h. The cells were broken down using Cell Signaling Technology's cell lysis buffer. This solution was prepared by combining protease inhibitors (Bio-Rad Laboratories, Inc., Hercules, CA, USA), phenylmethylsulfonyl fluoride (100 mmol/L), and Triton X-100 (0.5%, v/v). The protein content in the cell lysates was determined using a Bio-Rad protein assay kit. Cell lysate was loaded onto a polyacrylamide gel containing sodium dodecyl sulfate. A 5% (w/v) solution of nonfat milk in TBST was added to the proteins after they had been transferred to 0.45-*μ*m nitrocellulose membranes. Protein transfer was performed using 0.45-*μ*m nitrocellulose membranes. The membranes were then incubated with a 5% (w/v) solution of nonfat milk in TBST and incubated overnight at a temperature of 4°C with primary antibodies, including FAK, p-FAK, AKT, p-AKT, CD133, *β*-catenin, Nanog, and GADPH. The blots were exposed to secondary antibodies linked to horseradish peroxidase for a duration of 2 h. Each protein's quantitative value was determined using a chemiluminescence substrate from Bio-Rad. The intensity of the protein band was measured using the ImageJ software.

### 2.7. Statistical Analysis

The experimental data are presented as the mean, including the standard deviations (SDs). The analysis of variance (ANOVA) was used to assess multiple comparisons, and Scheffé's test was used for post hoc multiple means comparisons. The significance level was set at *p* < 0.05.

## 3. Results

### 3.1. Percent Yield and Appearance of PKE

The pumpkin pulp (Figure 1(a)) was pulverized and subjected to the extraction procedure.

The crude extract had a yield of 6.49% w/w when compared to dry weight. The acquired PKE exhibited a high viscosity and had a deep yellow hue.  Figure 1(b) illustrates the visual representation of PKE (Figure 1(b)).

### 3.2. Antioxidant Activities of the PKE

A recent study has demonstrated the antioxidant capabilities of PKE by subjected to testing against the DPPH radical [26]. This is a conventional method for assessing the radical scavenging activity of free radical compounds.  Figure 1(c) illustrates the DPPH scavenging activity of PKE at an IC_50_ value of 4.68 mg/mL, with the inhibition of DPPH radicals ranging from 25.20 ± 0.30 to 82.41 ± 1.23%. The IC_50_ value of ascorbic acid was 33.01 *μ*g/mL, which is lower than the PKE (Figure 1(d)). Based on the findings, PKE has a lower antioxidant capacity than the known antioxidant, ascorbic acid.

**Figure 2:**
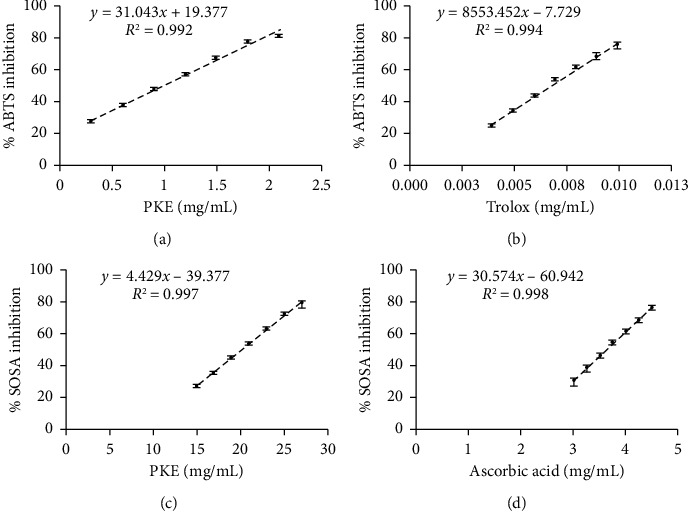
Total antioxidant activity was examined by using the ABTS radical scavenging activity and SOSA determination. The discoloration of ABTS radical cation by (a) PKE and (b) Trolox was investigated. The inhibition of SOSA by (c) PKE and (d) ascorbic acid was examined. A scatter plot was generated to demonstrate the relationship between sample concentration and inhibition percentage. Data are shown as the mean ± SD (*n* = 3).

The assessment of the total antioxidant activity was conducted using the decolorization of ABTS∙+ [26]. The findings were expressed as a percentage of the inhibition of the ABTS∙+ radical. PKE reduced the absorbance of the ABTS∙+ radical in a dose-dependent manner (Figure 2(a)). The IC_50_ values for PKE and Trolox (the standard compound, Figure 2(b)) were found to be 0.973 ± 0.014 mg/mL and 0.0067 ± 0.0001 mg/mL, respectively.

To explore PKE's antioxidant properties against superoxide anion, SOSA was quantified [26]. The IC_50_ value for the inhibition of SOSA by PKE was determined to be 20.18 ± 0.11 mg/mL, as shown in Figure 2(c). Nevertheless, the IC_50_ value of PKE was found to be higher than that of ascorbic acid (3.63 ± 0.03 mg/mL), as shown in Figure 2(d).

### 3.3. Total Phenolic Contents of the PKE

It has been reported that the pulp of *Cucurbita moschata* contains phenolic acids [13, 21]. There is a possibility that phenolic chemicals are directly involved in the antioxidant action. Therefore, the Folin–Ciocalteu test was employed to measure the total phenolic content of our PKE, with gallic acid serving as the standard compound for comparison [26]. As measured by GAE, the total phenolic content of PKE per gram extract was 141.211 ± 11.443.

### 3.4. Phytochemistry Analysis

In response to the antioxidant activity and other biological activities of PKE, the phytochemical composition was investigated. We assessed the phytochemical composition of PKE, encompassing alkaloids, anthraquinones, glycosides, tannins, xanthones, triterpenes, and steroids [27]. The examination of PKE's phytochemistry revealed the existence of alkaloids, glycosides, xanthones, triterpenes, and steroids (Table 1). High concentrations of xanthones were observed. Moderate quantities of triterpenes, steroids, and glycosides were found, although alkaloids were discovered at low concentrations. The analysis did not detect the presence of anthraquinones and tannins.

### 3.5. Qualitative Analysis of Bioactive Compounds by Using HPLC

The previously identified compounds, ɑ-tocopherol, riboflavin, protocatechuic acid, *β*-carotene, and luteolin [13, 19, 21–23], were analyzed in PKE. These essential nutritional elements and bioactive compounds were found abundantly in *Cucurbita moschata* pulp. Previous reports have indicated their strong antioxidant properties [31–33], increased migration [34–36], and stemness [37–39]. Our study found that *α*-tocopherol exhibited the highest concentration (126.72 ± 0.69 mg/L), followed by riboflavin, protocatechuic acid, and *β*-carotene. Luteolin exhibited the lowest concentration at 2.95 ± 0.01 mg/L (Table 2).

### 3.6. Effect of PKE on the Biological Activity of Human Skin Cells

#### 3.6.1. Effect of PKE on the Viability and Death of Human Keratinocyte and Human Primary Skin Fibroblast Cells

**Figure 3:**
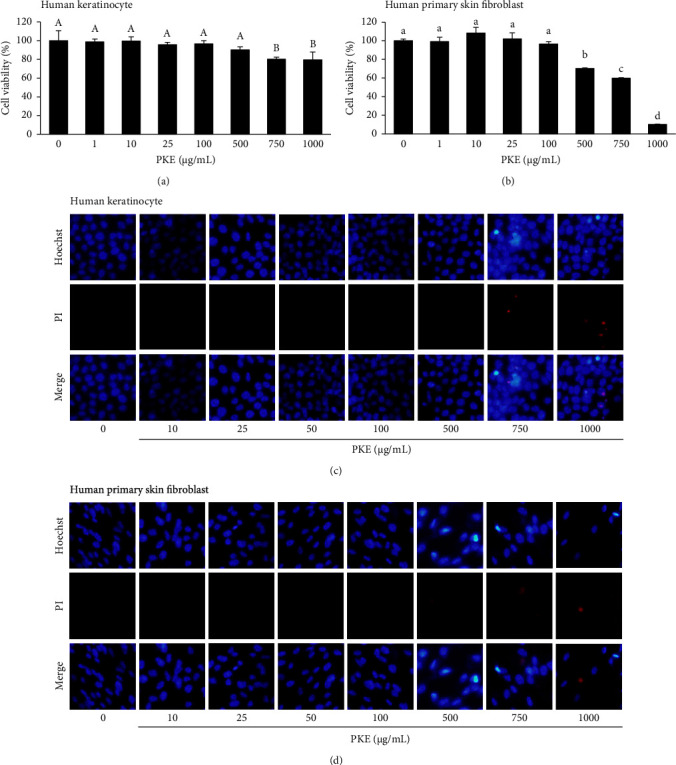
Effect of PKE on viability and death of human keratinocyte and human primary skin fibroblast cells by using MTT assay. The cell viability of (a) HaCaT and (b) human primary skin fibroblast was assessed after treatment with PKE at concentrations ranging from 0 to 1000 *μ*g/mL for a duration of 24 h. Data are shown as the mean ± SD (*n* = 3). The mean bars with same letters at the top of error bars indicate no significant difference (*p* < 0.05). Both (c) HaCaT cells and (d) human primary skin fibroblasts were subjected to staining with Hoechst 33342 and propidium iodide (PI) after PKE treatment in order to assess the extent of cell death, including both apoptotic and necrotic processes.

To determine the wound healing and stemness of PKE, the nontoxicity concentration of PKE was first investigated in human skin cells. Human keratinocyte (HaCaT) and primary skin fibroblast cells were used as models to assess the extract's cytotoxicity. The MTT assay was performed to determine cell viability after exposing cells to PKE at concentrations ranging from 0 to 1000 *μ*g/mL for 24 h. The findings indicated that there was no significant difference in human keratinocyte cell viability between the untreated control and the PKE at dosages ranging from 0 to 500 *μ*g/mL (Figure 3(a)). Simultaneously, PKE at concentrations ranging from 0 to 100 *μ*g/mL did not exhibit any cytotoxic effects on human primary skin fibroblast cells (Figure 3(b)).

The cell viability test findings have been confirmed by the measurement of cell death induction using Hoechst 33342/PI labeling. Cells did not exhibit chromatin condensation or nuclear fragmentation at PKE doses ranging from 10 to 100 *μ*g/mL. This indicates that apoptotic and necrotic processes may be ruled out (Figures 3(c) and 3(d)). Therefore, nontoxic concentrations ranging from 0 to 100 *μ*g/mL were selected to be studied in further experiments.

#### 3.6.2. PKE Promotes Wound Healing in Human Keratinocyte and Human Primary Skin Fibroblast Cells

**Figure 4:**
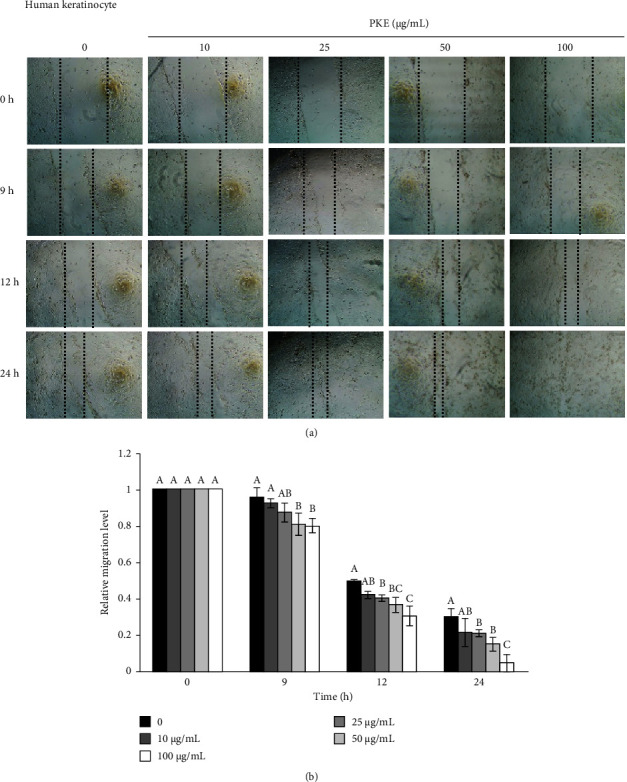
Effect of PKE on the migration of human keratinocyte HaCaT cells. Following a 24-h exposure to PKE at concentrations ranging from 0 to 100 *μ*g/mL, the migratory capacity of HaCaT cells was assessed using a scratch wound healing assay. (a) The migration of cells was captured with phase-contrast images (10×) at 0, 9, 12, and 24 h. In addition, (b) the relative migration of cells at each time point in relation to 0 h was calculated. Data are shown as the mean ± SD (*n* = 3). Within the same time of treatment, the mean bars with same letters at the top of error bars indicate no significant difference (*p* < 0.05).

**Figure 5:**
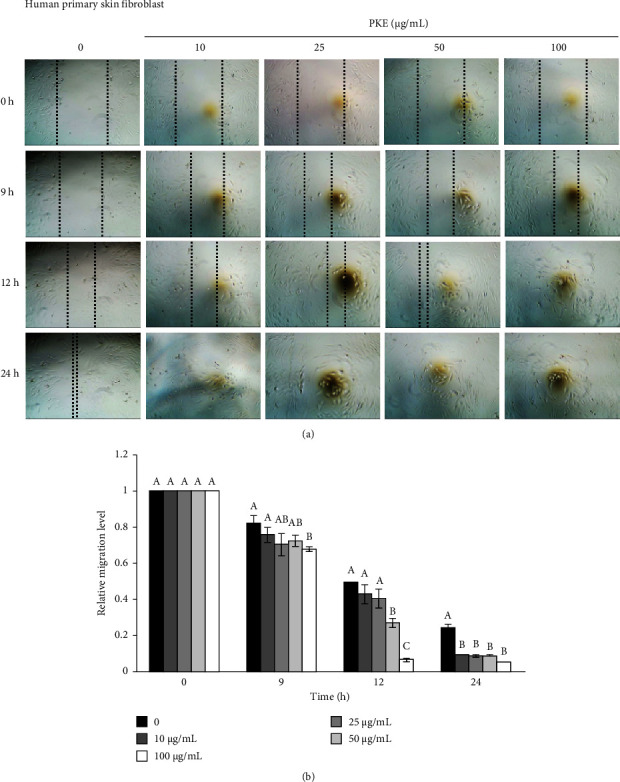
Effect of PKE on the migration of human primary skin fibroblast cells. Following a 24-h exposure to PKE at concentrations ranging from 0 to 100 *μ*g/mL, the migratory capacity of human primary skin fibroblast cells was assessed using a scratch wound healing assay. (a) The migration of cells was captured with phase-contrast images (10×) at 0, 9, 12, and 24 h. (b) The relative migration of cells at each time point in relation to 0 h was calculated. Data are shown as the mean ± SD (*n* = 3). Within the same time of treatment, the mean bars with same letters at the top of error bars indicate no significant difference (*p* < 0.05).

The scratch wound healing assay was used to assess the migratory capacity of human keratinocyte HaCaT cells and human primary skin fibroblast cells after PKE treatment [26]. Following exposure to PKE for 9, 12, and 24 h, cell migration was evaluated.  Figure 4(a) demonstrates that the presence of PKE at concentrations ranging from 25 to 100 *μ*g/mL resulted in higher cell migration rates after 12 and 24 h than the initial 0-h measurement.  Figure 4(b) displays the relative migration levels of human keratinocyte cells, providing evidence that PKE treatment substantially increased the migration rates. At the same time, similar results were found in human primary skin fibroblast cells following treatment with PKE for 9–24 h (Figures 5(a) and 5(b)). The findings indicate that PKE has the potential to improve the healing of skin wounds by promoting the migration of human keratinocyte and human primary skin fibroblast cells.

#### 3.6.3. PKE Enhances Cell Migration Markers

**Figure 6:**
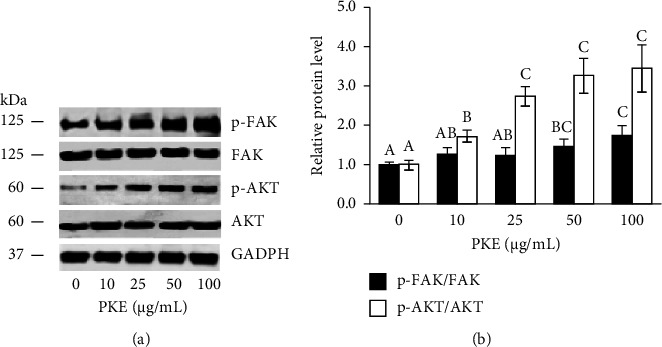
Protein expression of the FAK/AKT signaling pathway. (a) HaCaT cells were treated with PKE for 24 h and the expression of FAK, phosphorylated FAK, AKT, and phosphorylated AKT was measured using Western blotting. (b) Protein levels in treated HaCaT cells were compared to protein levels in the control group and represented as the relative protein level. Data are shown as the mean ± SD (*n* = 3). Within each protein group, the mean bars with same letters at the top of error bars indicate no significant difference (*p* < 0.05).

Evidence has shown that the FAK/AKT signal pathway results in subsequent effects, such as increased cell migration [10, 11]. We used Western blotting to identify the impact of PKE treatment on motility-regulating proteins, including FAK, phosphorylated FAK, AKT, and phosphorylated AKT. Figures 6(a) and 6(b) illustrate a significant increase in FAK activation when cells were exposed to a concentration of 50–100 *μ*g/mL of PKE for a duration of 24 h. This was followed by a dramatic increase in the phosphorylation of downstream AKT proteins (Figures 6(a) and 6(b) and supporting Figure 1). These results provide further evidence that PKE treatment promotes cell activity related to migration via a mechanism that relies on FAK/AKT.

#### 3.6.4. PKE Promotes Stem Cell–Like Phenotypes in Human Keratinocyte Cells

**Figure 7:**
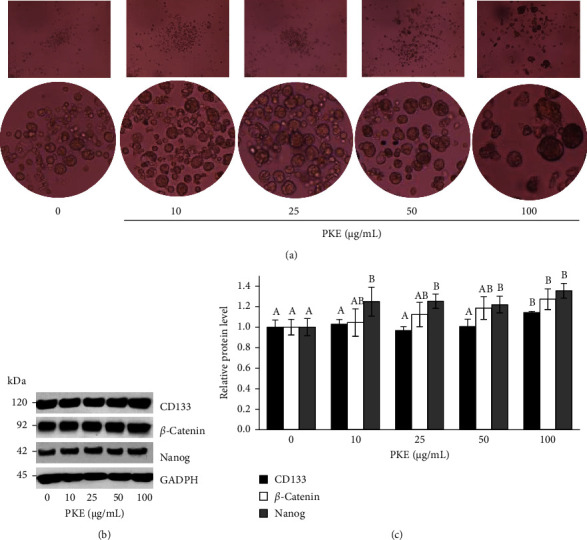
Effect of PKE on stem cell–like phenotype and expression of stemness regulatory protein in human keratinocyte cells. Following treatment with PKE 0–100 *μ*g/mL for 3 days, HaCaT cells were subjected to spheroid formation assay as a single-cell suspension. (a) 4 x phase-contrast images of spheroids at day 14 were captured. (b) The expression of CD133, *β*-catenin, and Nanog was measured using Western blotting. (c) Protein levels in treated HaCaT cells were compared to protein levels in the control group and represented as the relative protein level. Data are shown as the mean ± SD (*n* = 3). Within each protein group, the mean bars with same letters at the top of error bars indicate no significant difference (*p* < 0.05).

Keratinocyte stem cells are often assessed by their ability to generate three-dimensional spheroids, a widely accepted approach for discerning stem cell–like characteristics [30]. The spheroid forms were shown to be more abundant in the PKE-treated cells in comparison to the untreated cells (Figure 7(a)). These findings suggest that PKE could improve the characteristics of stem cells in human keratinocyte cells.

CD133 is often used as a marker to ascertain the identification of stem cells. Cells expressing CD133 were shown to have a greater ability to grow into spheroids [30]. Cells were treated with PKE (0–100 *μ*g/mL) for 24 h in order to evaluate the quantity of CD133 using Western blotting. It was shown that PKE enhances the expression level of CD133 (Figure 7(b) and supporting Figure 2). The findings show that PKE enhances the stem cell properties seen in human keratinocyte cells.

#### 3.6.5. PKE Promotes Stemness Regulatory Proteins in Human Keratinocyte Cells

To provide more evidence of the inductive activity of stem cells, we assessed the expression of the upstream protein signaling molecule *β*-catenin and Nanog, a transcription factor that enhances self-renewal [8, 9]. The expression of both *β*-catenin and Nanog was shown to be upregulated by PKE (Figures 7(b) and 7(c) and supporting Figure 2). These data suggest that PKE enhances the stem cell–like characteristics via increasing the expression of *β*-catenin and Nanog.

## 4. Discussion

The skin serves as the primary barrier protecting the body from many external factors, including physical, chemical, and biological agents. After sustaining an injury, the body initiates a sequence of consecutive processes to repair the damage, ultimately leading to the development of a scar. These processes include hemostasis, inflammation, proliferation, and remodeling [1]. The first cell type to migrate to the wound site is the keratinocyte, which subsequently initiates re-epithelialization, enhances cell proliferation, and ultimately occupies the wound area. In addition, keratinocytes stimulate fibroblasts to secrete growth factors that regulate the proliferation of fibroblasts [2]. Our investigation showed that PKE is safe for human skin cells, including keratinocytes and fibroblasts (Figure 3). Sublethal doses of PKE enhance the migration of both keratinocytes and fibroblasts (Figures 4 and 5). In vitro research has shown that when migrating keratinocytes encounter a damaged monolayer during epidermal wound healing, there is an increase in the expression of FAK [11]. Based on the existing evidence, it may be inferred that FAK controls cellular morphology and movement via several subsequent pathways, such as AKT [11]. PKE was shown to promote the expression of the active form of FAK and AKT in keratinocytes (Figure 6). The findings indicate that PKE is simultaneously safe and enhances the migratory impact in human skin cells via activating the FAK/AKT signaling pathway.

Stem cells have the capacity to overcome the limitations of current wound care therapies. This phenomenon occurs because stem cells facilitate accelerated tissue regeneration during the wound-healing process [5, 6]. Keratinocyte stem cells play a crucial role in maintaining the balance and healing processes of the skin [5]. The findings of our research demonstrate that PKE enhances the phenotypic characteristics of HaCaT keratinocyte stem cells by promoting the development of spheroid cells (Figure 7(a)) and increasing the level of the CD133 stem cell marker (Figures 7(b) and 7(c)). Studies have shown that CD133 controls cellular mechanisms associated with the maintenance and function of stem cells, as well as cell proliferation and differentiation [7]. It has been shown that an increase in the expression of CD133 is related to the formation of HaCaT keratinocyte stem cell spheroids, as reported in a study of the extract of *Grammatophyllum speciosum* [30]. In addition, PKE also led to an increase in the expression of *β*-catenin (Figures 7(b) and 7(c)). Augmentation of *β*-catenin was reported to upregulate Nanog in human mesenchymal stem cells (MSCs) and facilitate MSC self-renewal [40]. In our work, we observed an increase in Nanog (Figures 7(a) and 7(b)), which is a self-renewal factor that may maintain mouse embryonic stem cells (mESCs) and interacts with several other critical factors involved in stem cell regulation of pluripotency [9, 40]. Our findings suggest that the wound-healing potential of PKE may be attributed to the augmentation of stemness in keratinocyte stem cells, advocating for the emerging practice of using stem cells in the treatment of skin wounds [6].

Excessive levels of ROS lead to injury to keratinocyte cells and hinder the process of wound healing [1]. Elevated amounts of ROS may lead to the degradation of ECM proteins and disrupt biological processes in keratinocytes and fibroblasts [3]. Wounds may undergo accelerated healing in an environment abundant in antioxidants [4]. Furthermore, several plants or plant-derived substances with notable antioxidant properties have shown wound-healing effects [4, 41]. PKE exhibited antioxidant activity against DPPH, ABTS, and superoxide anion (Figures 1 and 2). Previous studies have shown the antioxidant characteristics of *C. moschata* Duchesne [13, 21, 42]. Based on this research, the antioxidant action may be ascribed to compounds such as carotenoids and phenolic components. The analysis of the pulp of *C. moschata* Duchesne in our investigation confirms the earlier findings that PKE is abundant in *β*-carotene and protocatechuic acid (Table 2). *β*-Carotene and protocatechuic acid have been recognized as potent antioxidants capable of neutralizing DPPH, ABTS, and superoxide anion [31, 32]. *α*-Tocopherol (vitamin E), a potent antioxidant [33], is also found in PKE (Table 2). Similar to the previous investigation, *α*-tocopherol was identified in pumpkin extract [19]. Therefore, the migratory influence of PKE on human keratinocyte and fibroblast migration may be enhanced by antioxidant substances, as suggested in the previous reports [4, 41].

Earlier findings indicated that the phytochemicals found in PKE (Tables 1 and 2), for example, protocatechuic acid, *β*-carotene, *ɑ*-tocopherol, and xanthones, have been shown to enhance cellular motility and stemness [34–39, 43]. Protocatechuic acid derived from *Alpinia oxyphylla* promoted the movement of human adipose tissue–derived stromal cells (hADSCs) in an in vitro study [34]. Furthermore, protocatechuic acid promotes the migration of neuronal RSC96 Schwann cells by activating the MAPK pathway [35]. Under high-glucose and hypoxic conditions, the migratory activity of endothelial progenitor cells (EPCs) was enhanced by ɑ-tocopherol [36]. The presence of xanthones in PKE may have the capacity to increase the migratory effect. A recent study has shown that xanthones increase the migration rate of skin fibroblasts [43]. The effects of protocatechuic acid, *β*-carotene, and ɑ-tocopherol on stem cells have been previously reported [37–39]. Protocatechuic acid has been shown to enhance the proliferation of neural stem cells (NSCs) while reducing the levels of ROS [37]. *β*-Carotene enhanced the process of differentiation in mouse ciliary epithelium–derived MSCs, leading to the development of specialized retinal cells [39]. In spermatogonial stem cells (SSCs), *α*-tocopherol has been reported to induce their proliferation in vitro and in vivo [38]. Thus, the phytochemical components found in PKE may promote the migration of human skin cells and the stemness of keratinocyte stem cells.

This study is limited to examining the effects of pumpkin pulp extract on human skin cells. In order to understand the molecular mechanism by which compounds stimulate migratory and stemness-regulating proteins, further investigation is required on the effect of phytochemicals present in PKE or their combination effect. Furthermore, topical application of PKE formulation in human or animal studies should be conducted to assess its safety and efficacy.

## 5. Conclusions

The enhanced migration rate of keratinocyte and fibroblast cells exhibited the wound-healing advantages of PKE. The potentiation of stemness in keratinocyte stem cells via *β*-catenin and Nanog activation could be a novel application of PKE in wound-healing properties. The compound's antioxidant capabilities provide a possible explanation for PKE's wound-healing activities. The presence of alkaloids, glycosides, xanthones, triterpenes, and steroids was identified through phytochemistry analysis. Additionally, bioactive compounds such as ɑ-tocopherol, riboflavin, protocatechuic acid, *β*-carotene, and luteolin were detected using HPLC. Results from this study suggest that the major compound in this extract, *α*-tocopherol, along with other detected compounds, plays a role in the antioxidant activity, stemness enhancement, and wound-healing activity of PKE. The discoveries might facilitate the identification of significant natural resources that have the potential to be used in wound healing via a clearly observable molecular process.

## Figures and Tables

**Table 1:** Phytochemistry components of PKE.

Phytochemistry components	Remarks
Alkaloids	+
Anthraquinones	—
Glycosides	Fructose +
Tannins	—
Xanthones	+++
Triterpenes	++
Steroids	++

*Note:* “+++” denotes high concentration, “++” denotes moderate concentration, “+” denotes low concentration, and “—” denotes not detected. Phytochemistry components of ethanolic extract derived from *C. moschata* fruit pulp (PKE).

**Table 2:** Main composition of PKE obtained by using HPLC.

Phytochemistry components	Concentration (mg/L)
*α*-Tocopherol	126.72 ± 0.69
Riboflavin	81.77 ± 0.90
Protocatechuic acid	37.71 ± 0.24
*β*-Carotene	27.45 ± 0.15
Luteolin	2.95 ± 0.01

*Note:* Main composition of PKE obtained by using high-performance liquid chromatography (HPLC).

## Data Availability

The data that support the findings of this study are available on request from the corresponding author.

## Supporting Information

Supporting Figure 1: The images of original uncropped Western blots of FAK, phosphorylated FAK, AKT, phosphorylated AKT, and GADPH. Supporting Figure 2: The images of original uncropped Western blots of CD133, *β*-catenin, Nanog, and GADPH.
