# Supplementary material for: In Vitro Evaluation of Wound Healing, Stemness Potentiation, Antioxidant Activity, and Phytochemical Profile of Cucurbita moschata Duchesne Fruit Pulp Ethanolic Extract
Source: Adv Pharmacol Pharm Sci. 2024 Oct 28;2024:9288481. doi: 10.1155/2024/9288481 (PMC11535185; doi:10.1155/2024/9288481)
Supplement: Supporting Information — Additional supporting information can be found online in the Supporting Information section. [file 9288481.f1.docx]

**Supplement materials**

| **Proteins** | **Blots** |
| --- | --- |
| **p-FAK** | 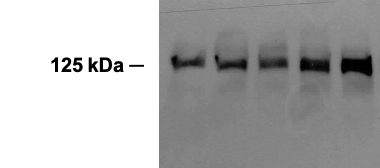 |
| **FAK** | 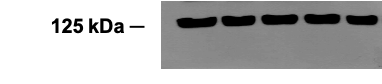 |
| **p-AKT** | 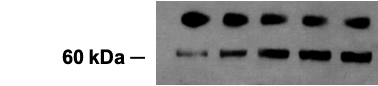 |
| **AKT** | 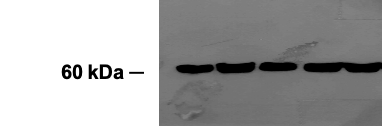 |
| **GADPH** | 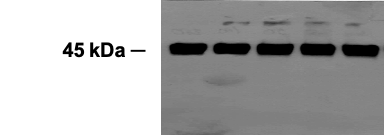 |

**Supplementary Figure 1** Images of original uncropped Western blots used for preparation of Figure 6a.

| **Proteins** | **Blots** |
| --- | --- |
| **CD133** | 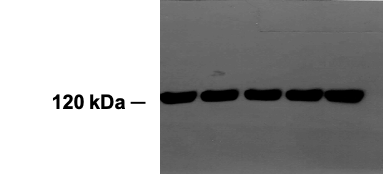 |
| **β-catenin** | 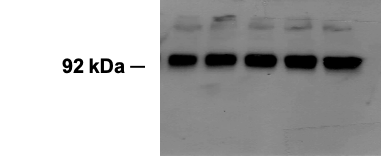 |
| **Nanog** | 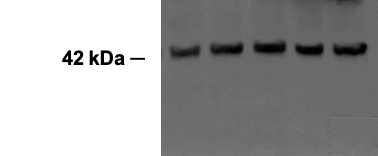 |
| **GADPH** | 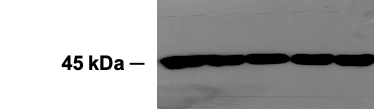 |

**Supplementary Figure 2** Images of original uncropped Western blots used for preparation of Figure 7b.
